# Pan-Cancer Analysis Reveals Differential Susceptibility of Bidirectional Gene Promoters to DNA Methylation, Somatic Mutations, and Copy Number Alterations

**DOI:** 10.3390/ijms19082296

**Published:** 2018-08-05

**Authors:** Jeffrey A. Thompson, Brock C. Christensen, Carmen J. Marsit

**Affiliations:** 1 Department of Biostatistics, University of Kansas Medical Center, Kansas City, KS 66160, USA; 2 Department of Epidemiology; Geisel School of Medicine at Dartmouth College, Hanover, NH 03755, USA; Brock.C.Christensen@dartmouth.edu; 3 Department of Molecular and Systems Biology, Geisel School of Medicine at Dartmouth College, Hanover, NH 03755, USA; 4 Department of Community and Family Medicine, Geisel School of Medicine at Dartmouth College, Hanover, NH 03755, USA; 5 Department of Environmental Health, Rollins School of Public Health at Emory University, Atlanta, GA 30322, USA; Carmen.J.Marsit@emory.edu

**Keywords:** pan-cancer, bidirectional promoters, head-to-head genes

## Abstract

Bidirectional gene promoters affect the transcription of two genes, leading to the hypothesis that they should exhibit protection against genetic or epigenetic changes in cancer. Therefore, they provide an excellent opportunity to learn about promoter susceptibility to somatic alteration in tumors. We tested this hypothesis using data from genome-scale DNA methylation (14 cancer types), simple somatic mutation (10 cancer types), and copy number variation profiling (14 cancer types). For DNA methylation, the difference in rank differential methylation between tumor and tumor-adjacent normal matched samples based on promoter type was tested by the Wilcoxon rank sum test. Logistic regression was used to compare differences in simple somatic mutations. For copy number alteration, a mixed effects logistic regression model was used. The change in methylation between non-diseased tissues and their tumor counterparts was significantly greater in single compared to bidirectional promoters across all 14 cancer types examined. Similarly, the extent of copy number alteration was greater in single gene compared to bidirectional promoters for all 14 cancer types. Furthermore, among 10 cancer types with available simple somatic mutation data, bidirectional promoters were slightly more susceptible. These results suggest that selective pressures related with specific functional impacts during carcinogenesis drive the susceptibility of promoter regions to somatic alteration.

## 1. Introduction

Approximately 10% of human genes have bidirectional promoters [1,2], where a promoter region is shared between two genes on opposite strands and initiates transcription in both directions. In practice, the definition of bidirectional promoters that is typically used does not include actual bidirectional function. Instead, promoters are said to be bidirectional if they lie between genes on opposite strands whose transcription start sites (TSSs) are within 1000 bp of each other [2,3,4]. This definition is somewhat arbitrary, based on the first large characterization of the arrangement following the completion of the human genome [4]. Nevertheless, it has proven useful in subsequent studies, through which genes with this promoter arrangement have been found to be co-expressed in many contexts [2,5,6,7]. Using this definition, it has been shown that genes with bidirectional promoters are enriched for genes implicated in cancers, including *BRCA1* and *TP53* [6,7]. Nevertheless, it appears that bidirectional transcription is initiated at many, if not most promoters [2,3]. In most cases, this transcription is paused or aborted in one direction through channels that are not entirely clear but likely include nucleosome positioning, histone modifications, and other regulatory mechanisms [3]. 

Bidirectional promoters can be classified into two types: (1) a bidirectional promoter between two genes that code for protein called coding/coding (C/C) bidirectional promoters; and (2) a bidirectional promoter with one protein coding and one noncoding gene called coding/noncoding (C/N) bidirectional promoters. The incomplete characterization of functional noncoding transcripts puts noncoding/noncoding bidirectional promoters outside the scope of this work. Bidirectional promoters are also enriched for CpG islands, with approximately 80% of these promoters containing a CpG island [8], compared to approximately 60–70% for promoters overall [9]. Functionally, genes with the bidirectional promoters are enriched in biological processes related to chromatin maintenance, including nucleosome assembly, chromatin assembly or disassembly, DNA repair, and chromatin remodeling, as well as a number of metabolic and other processes [1,8].

**Figure 1:**
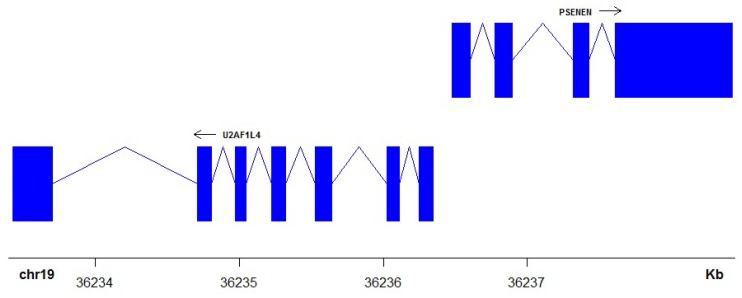
Bidirectional promoter arrangement of PSENEN and U2AF1L4 on chromosome 19. This gene pair has a validated bidirectional promoter that coordinates the expression of these two genes. Arrows indicate direction of transcription, blue boxes indicate exons, and physical position on chromosome 19 is shown in kilobases (Kb).

Given that in many contexts bidirectional promoters directly affect the transcription of two genes, genetic mutations or epigenetic changes that affect the promoter region could have twice the impact they might have in single gene promoters. These impacts could be particularly deleterious, given the enrichment for important functions that genes with this arrangement exhibit. Therefore, it has been suggested that adverse changes might be selected against more robustly than in single gene promoters [7]. An example of a gene pair with a confirmed bidirectional promoter arrangement (involving coordinated expression of both genes), is shown in Figure 1. *PSENEN* and *U2AF1L4* are co-expressed from a small, common promoter in multiple cell types, and mutations in the promoter affect the transcription of both genes [10]. Nevertheless, these genes have divergent functions, with *PSENEN* a component of the γ-secretase complex required for Notch signaling [11], and *U2AF1L4* a pre-mRNA splicing factor [10]. Thus, mutations affecting this promoter might disrupt entirely different processes. Similarly, the bidirectional function of the shared promoter of *SIRT3* and *PSMD13* has been confirmed [12]. However, *SIRT3* regulates the mitochondrial response to stress [13] and *PSMD13* plays a role is degrading abnormal proteins [12]. To date, most putative bidirectional promoters have not been lab validated; however, a recent study demonstrated that the majority of these gene arrangements are conserved between human and mouse genomes and also display similar patterns of expression [14]. This suggests that the bidirectional arrangement is not random and may play an important role. In fact, although genes with bidirectional promoters are enriched for certain functions overall, conservation of the promoters does not appear to be strongly associated to shared functions between gene pairs themselves [15], suggesting even more strongly that changes to these promoters would be very disruptive.

The only study we are aware of to test the hypothesis that alterations to bidirectional promoters are selected against in carcinogenesis investigated it in the case of DNA methylation changes in cancer [7]. That work suggested that genes with bi-directional promoters are not protected from silencing through *de novo* methylation in cancer. However, the study used an unpublished dataset of an unknown sample size and relied on methylated CpG island amplification/representational difference analysis (MCA/RDA) to identify differentially methylated CpG islands, a technique that does not have the broad coverage and sensitivity of more recent methylation microarrays. Although we are not aware of a study of somatic mutation in bidirectional promoters *per se*, somatic mutation density as it relates to chromatin accessibility has been studied [16]. It was found that highly accessible chromatin, in the form of DNAse I hypersensitive sites (DHSs), tended to have a lower somatic mutation density across multiple cancers. Given that DHSs are enriched in promoters [17] and that bidirectional promoters control the activation of two genes, and thus may be active more frequently, it might be expected that bidirectional promoter regions tend to be more accessible and therefore have a lower somatic mutation density.

To test the hypothesis that bidirectional promoters are protected from somatic alteration in the process of carcinogenesis, we compared differential methylation across 14 cancer types and 710 matched samples, somatic mutation across 10 cancer types and 2473 samples, and copy number alteration across 14 cancer types and 6763 samples in C/C and C/N bidirectional gene promoters to single gene promoters. This work comprises the largest and most comprehensive examination of differential methylation, somatic mutation, and copy number alteration in bidirectional promoters in cancer to date.

## 2. Results

### 2.1. DNA Methylation

**Figure 2:**
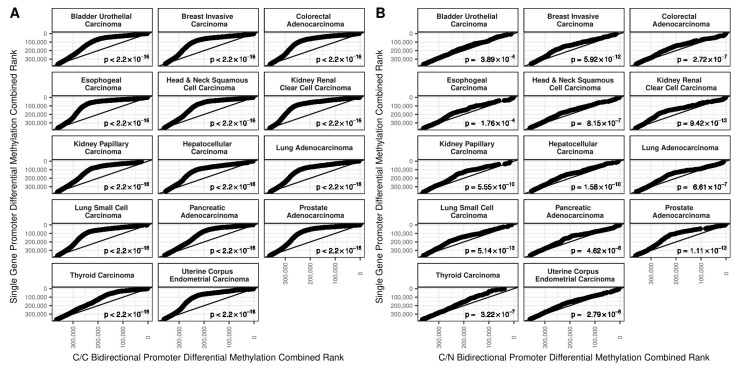
Quantile-quantile plots demonstrating degree of differential methylation in 17,639 single gene vs. 725 C/C (**A**) and 135 C/N (**B**) bidirectional promoters. At every quantile of rank differential methylation for bidirectional promoters, the rank of differential methylation for single gene promoters was always lower. This means that the single gene promoters were consistently more differentially methylated than bidirectional gene promoters for both bidirectional promoter types. For every cancer, these results were statistically significant.

We tested the hypothesis that the mean rank of differential methylation between tumor and tumor-adjacent normal samples is different between single gene and either C/C or C/N bidirectional promoters to indicate if a greater change in methylation was observed in one promoter type compared to the other using a two-sided Wilcoxon rank sum test. Overall and irrespective of promoter type, there is a tendency towards increased methylation of promoters in tumor samples compared to tumor-adjacent normal tissue. However, for each of the 14 cancer types examined (Table 1), the change in methylation was statistically significantly greater for single gene promoters compared to either C/C or C/N bidirectional promoters. This is visualized for all cancer types considered in Figure 2 as a series of quantile-quantile plots. These plots show that at any given quantile, the differential methylation is greater (i.e., lower rank) in the single gene compared to either the C/C or C/N bidirectional promoters, although the effect is less pronounced in the C/N bidirectional promoters.

**Figure 3:**
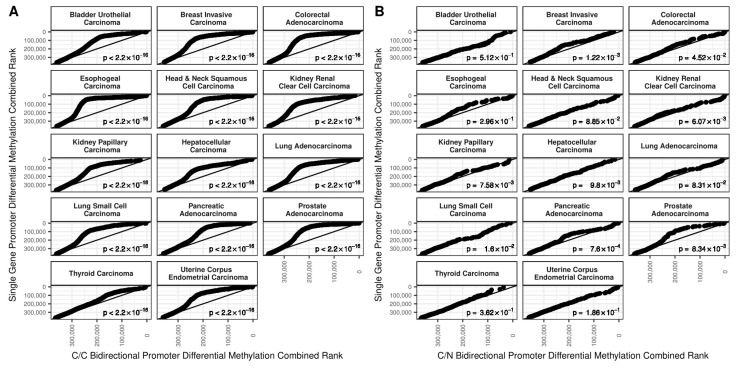
Quantile-quantile plots demonstrating degree of differential methylation in 5003 single gene vs. 657 C/C (**A**) and 97 C/N (**B**) bidirectional promoters restricted to CpG islands. For C/C bidirectional promoters, at every quantile of rank differential methylation, the rank of differential methylation for single gene promoters was always lower (i.e., greater differential methylation). For C/C bidirectional promoters, all results were statistically significant. For C/N bidirectional promoters, this trend mostly continued, but it was much weaker and was not apparent for all cancers.

To control for the effect of G/C content on the results, we restricted the promoter regions to only those intersecting CpG islands as annotated in the UCSC Genome Browser [18]. The results are shown in Figure 3. For C/C bidirectional promoters the results were essentially the same. For C/N bidirectional promoters the overall trend was the same, but the difference was much less apparent, and the overall difference was not always statistically significant.

### 2.2. Simple Somatic Mutations

**Figure 4:**
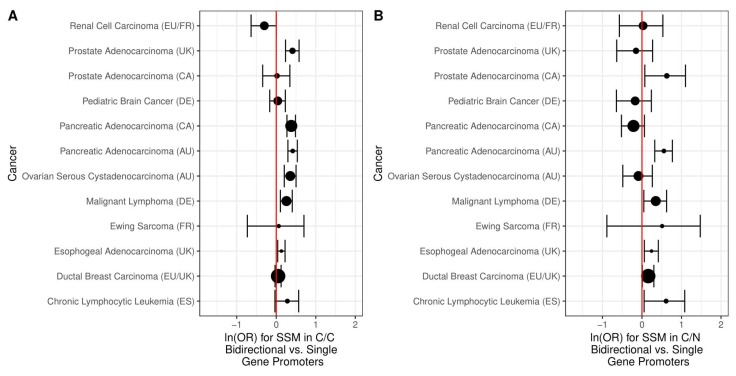
The log odds of simple somatic mutations in bidirectional vs. single gene promoters. The size of the points indicates the relative sample size and 95% confidence intervals are shown. (**A**) For C/C bidirectional promoters, there were somewhat higher odds of SSMs compared to single gene promoters for most cancers (the only exception was renal cell carcinoma). These results were statistically significant in six of the datasets; (**B**) For C/N bidirectional promoters, there were higher odds of SSMs in 7 of the 12 datasets and 6 of these were statistically significant.

**Figure 5:**
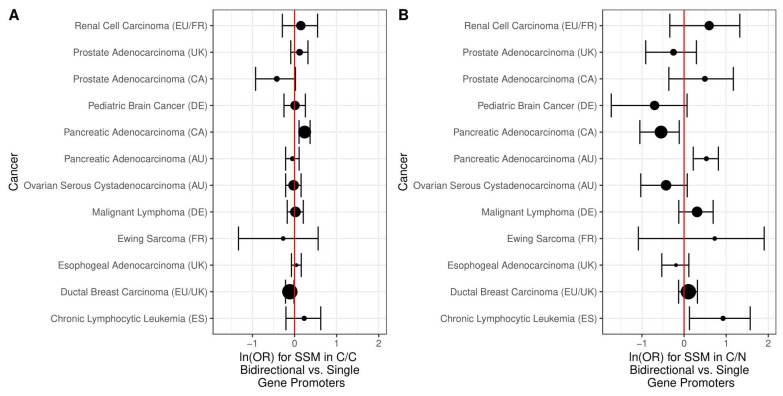
The log odds of simple somatic mutations in CpG islands in bidirectional vs. single gene promoters. (**A**) For C/C bidirectional promoters, after subsetting to CpG islands, the only statistically significantly greater odds of SSMs remaining is for the Canadian pancreatic cancer dataset; (**B**) For C/N bidirectional promoters, after subsetting to CpG islands, the only statistically significantly greater odds of SSMs is the Australian pancreatic cancer dataset and the leukemia dataset. Furthermore, the Canadian pancreatic dataset has significantly reduced odds of SSMs compared to single gene promoters.

We examined the odds of simple somatic mutations (SSMs) occurring in bidirectional vs. single gene promoters using 12 datasets covering 10 cancer types (Table 2). For most cancers, there were somewhat elevated odds of SSMs to occur in C/C bidirectional promoters compared to single gene promoters and about half of the cancers for C/N bidirectional promoters (Figure 4). In the case of C/C bidirectional promoters, there were statistically significant increased odds of SSMs for 1 of the 2 prostate cancer data sets, both pancreatic cancer data sets, as well as the ovarian, lymphoma, and esophogeal cancer datasets. For C/N bidirectional promoters, there were statistically significantly increased odds of SSMs for the other prostate cancer data set, one of the pancreatic cancer datasets, as well as the lymphoma, esophogeal, breast, and chronic lymphocytic leukemia datasets. Given that bidirectional promoters are known to be enriched for CpG islands, we considered that mutations may be driven by sequence differences. Therefore, we also determined the odds of somatic mutations for only the sections of bidirectional or single gene promoters that intersect CpG islands (Figure 5). Naturally, this reduced our power for detecting effects, widening the confidence intervals, but for most cancers the increased odds of SSMs goes away when considering only the portion of promoters that intersect CpG islands. The only statistically significantly increased odds for C/C bidirectional promoters remaining was for the Canadian pancreatic cancer datasets, and for C/N bidirectional promoters the Australian pancreatic cancer dataset and the leukemia dataset. Also, for C/N bidirectional promoters, there were significantly decreased odds of an SSM relative to single gene promoters in the Canadian pancreatic cancer dataset.

### 2.3. Somatic Copy Number Alterations

**Figure 6:**
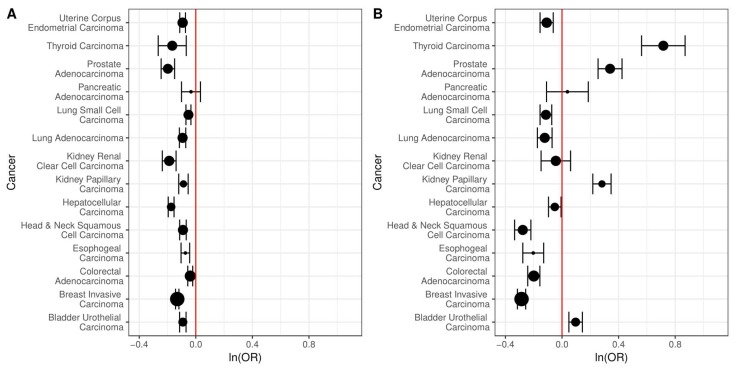
The log odds of intersecting regions of copy number alteration in bidirectional vs. single gene promoters. The size of the points indicates the relative sample size and 95% confidence intervals are shown. (**A**) The odds of intersecting regions of copy number alteration are lower for the 725 C/C bidirectional compared to 17,639 single gene promoters, across all 14 cancers. These results are statistically significant for 13 out of 14 cancers; (**B**) The odds of intersecting regions of copy number alteration are lower for the 135 C/N bidirectional compared to single gene promoters, across 9/14 cancers. The results are statistically significant in 12 out of 14 cancers.

We next investigated the association of copy number alteration to bidirectional vs. single gene promoters using the same 14 cancer types used to study changes in DNA methylation (Table 1). We compared the odds of a region of copy number variation intersecting a C/C or C/N bidirectional promoter to the odds for a single gene promoter. For all cancers, there was a reduced odds of somatic copy number change for C/C bidirectional promoters compared to single gene promoters, which was also true for 9/14 C/N bidirectional promoters. In most cases, the results were statistically significant (Figure 6). 

Past work has also suggested an association between copy number alteration and chromosomal fragile sites, which tend to break more frequently under the stress of replication [19]. Therefore, we examined bidirectional promoters for enrichment in chromosomal fragile sites compared to single gene promoters using a list of sites compiled in a prior study [20]. C/C bidirectional promoters have slightly greater odds of intersecting chromosomal fragile sites (OR 1.14, 95% CI [0.92, 1.39], *p* = 2.22 × 10^−1^), although the result is not statistically significant. C/N bidirectional promoters have even greater odds of intersecting chromosomal fragile sites (OR 1.48, 95% CI [0.94, 2.27], *p* = 6.95 × 10^−2^), although it is still not statistically significant.

**Figure 7:**
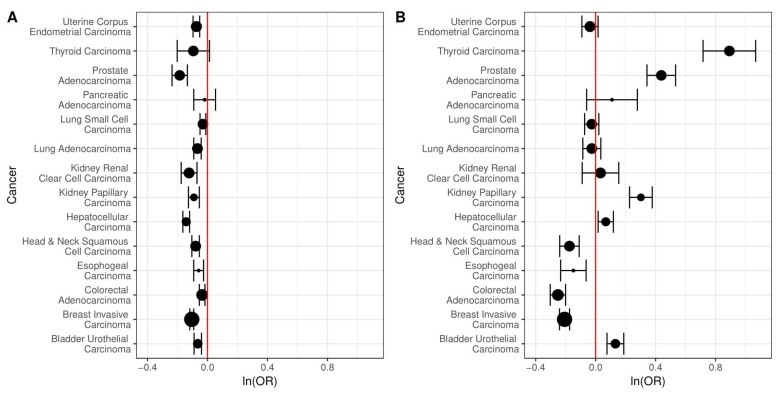
The log odds of intersecting regions of copy number alteration in bidirectional vs. single gene promoters, restricted to CpG islands. The size of the points indicates the relative sample size and 95% confidence intervals are shown. (**A**) The odds of intersecting regions of copy number alteration are lower for the 657 C/C bidirectional compared to 5003 single gene promoters, across all 14 cancers. These results are statistically significant for 12 out of 14 cancers; (**B**) For the 97 C/N bidirectional compared to single gene promoters, the odds of intersecting regions of copy number alteration are lower for only half the cancers. The results are significant in 8/14 cancers.

It has also been shown that the breakage frequency of chromosomal fragile sites is negatively correlated with CpG island density. Given that bidirectional promoters tend to have a higher percentage of CpG islands than single gene promoters, we compared the odds of a region of copy number variation intersecting a C/C or C/N bidirectional promoter to the odds for a single gene promoter only for promoters with CpG islands. For all cancers, there was a reduced odds of somatic copy number change for C/C bidirectional promoters compared to single gene promoters, even after restricting to only those regions with CpG islands (Figure 7), and most of these results were statistically significant. For C/N bidirectional promoters, there were statistically significant reduced odds of copy number change only for head and neck, esophogeal, colorectal, and breast cancer. There were significantly increased odds for thyroid, prostate, kidney papillary, liver, and bladder cancer. 

### 2.4. Cancer Genes

To extend our investigation, we also considered the enrichment of genes with bidirectional promoters vs. single gene promoters in the Catalog of Somatic Mutations in Cancer (COSMIC) cancer Gene Census [21], downloaded 13 September 2016. Genes with C/N bidirectional promoters were limited to the coding genes only, due to the lack of representation of noncoding genes in the cancer gene census. Overall, genes with C/C bidirectional promoters were not very enriched for known cancer genes (odds ratio 1.04, 95% CI [0.74, 1.43]. However, genes with C/N bidirectional promoters were enriched (not statistically significant) for known cancer genes relative to genes without bidirectional promoters (odds ratio 2.08 95% CI [0.88, 4.26], *p* = 6.45 × 10^−2^).

### 2.5. DNAse Hypersensitive Sites

To assess the relationship between accessible chromatin and promoter type, we compared the odds of C/C or C/N bidirectional promoters intersecting DNAse hypersensitive sites (DHSs) to those of single gene promoters intersecting DHSs. We obtained DHS data from the Roadmap Epigenomics Project for four tissues: breast, pancreas, ovary, and placenta [22,23]. In each case, bidirectional promoters were enriched for DHSs compared to single gene promoters, especially in the case of C/C bidirectional promoters (Table 3).

### 2.6. Functional Enrichment

To test for enrichment in biological processes in genes with C/C and C/N bidirectional promoters according to the Gene Ontology we used the online tool WEB-based GEne SeT AnaLysis Toolkit (WebGestalt) (http://www.webgestalt.org/) [24,25]. We used the genes we identified with C/C or C/N bidirectional promoters and single gene promoters as the background and restricted results to those with at least 5 genes and an adjusted *p*-value of at most 0.01. Consistent with previous work, we found that genes with C/C bidirectional promoters are enriched for chromatin organization, DNA repair genes, metabolic processes, and other functions previously identified (Table 4). Notably, genes with C/C bidirectional promoters are enriched for noncoding RNA metabolism and processing. Genes with C/N bidirectional promoters are not enriched for any biological process. 

## 3. Discussion

Past research indicated that bidirectional promoters may not have any particular protection against changes in methylation in cancer [7]. However, that work was limited in scope of sample size, cancer type, and data resolution compared with this study. In this work, we showed that in all 14 of the cancer types studied, there was a significantly greater change in methylation in single gene promoters compared to C/C and C/N bidirectional promoters. Even after controlling for differences in CpG frequency, this remained true for all C/C bidirectional promoters and many of the C/N bidirectional promoters. The overall trend in methylation change when it does exist is for an increase in the number of alleles methylated for loci in gene promoters, but this effect is observed mainly in single gene promoters. 

For several cancers, either C/C or C/N bidirectional promoters appear to be somewhat more susceptible to simple somatic mutations in cancer compared to single gene promoters, and our results suggest this result is driven by differences in the nucleotide content of the different promoter types. This result is somewhat surprising, because bidirectional promoters tend to be more active and accessible then single gene promoters, and previously, Polak, et al. linked such accessibility to a lower somatic point mutation density in cancer [16]. This could indicate that SSMs are being selected for in bidirectional promoters, at least in some cancers.

For most cancers, both C/C and C/N bidirectional promoters have lower odds of intersecting regions of somatic copy number variation than single gene promoters. After controlling for differences in G/C content, this result is only clear for C/C bidirectional promoters. This is interesting, because bidirectional promoters are more likely to intersect chromosomal fragile sites and thus may represent selection against change in copy number for regions with bidirectional promoters in most tumors, although this enrichment in chromosomal fragile sites was not statistically significant. However, not all chromosomal fragile sites break with the same probability. There is a negative correlation between breakage frequency and CpG island density [19]. Nevertheless, for C/C bidirectional promoters, the apparent protections against change in copy number persisted after controlling for CpG islands. The effect was less apparent for C/N bidirectional promoters, which also have a greater enrichment in chromosomal fragile sites. This may be partly explained by the noncoding gene in C/N bidirectional promoters. Noncoding genes have been shown to have an A/T rich nucleotide content, possibly leaving them more prone to chromosomal instability.

In the past, it has been noted that genes with bidirectional promoters include genes causally relevant to cancer. However, we did not find that genes with a C/C bidirectional arrangement had higher odds of being known causal cancer genes, with reference to COSMIC’s cancer gene census. Nevertheless, this may be the case for genes with C/N bidirectional promoters (although this includes only 8 genes, due to the smaller number of C/N bidirectional promoters identified overall and the result was not statistically significant). Concordant with past work, we did find that genes with C/C bidirectional promoters are enriched for chromatin organization, DNA repair, and metabolism functions (Table 4). Genes with C/N bidirectional promoters did not share any functions but did share some of the relative protection of C/C bidirectional promoters against change, at least in the case of DNA methylation and copy number alteration. This could support the hypothesis that the relative protection from change in DNA methylation is due to the bidirectional arrangement, rather than functional pathways that are being maintained, but the results are less clear for copy number alteration. 

This work comprised the largest analysis yet performed of genetic and epigenetic alterations to bidirectional promoters in cancer. We showed that genes with bidirectional promoters exhibit robust protections from changes in DNA methylation and copy number alteration, supporting the hypothesis that bidirectional promoters are protected, relative to other promoters, from these changes. Given that these results were only robust for C/C bidirectional promoters, it is not necessarily directly related to their bidirectional arrangement. It may be that genes with certain functions tend to be arranged in this way, and it is their function that causes the selection against change. In any case, these results suggest that the bidirectional promoter arrangement is enriched for genes that stay active, even in cancer, a finding which needs further confirmation and study. They further suggest that cancer cells require normal function from many genes with bidirectional promoters, which could lead to susceptibility to synthetic lethality involving some gene pairs that involve genes with bidirectional promoters. We also demonstrated that, in a number of cancers, genes with bidirectional promoters tend to accumulate a greater number of simple somatic mutations, possibly driven by their higher G/C nucleotide content. Furthermore, we defined a subclass of bidirectional promoters, which include one noncoding gene in the pair, and showed that in terms of their protection again change in cancer, they share some properties with other bidirectional promoters, although they are not enriched for the same functions that many other genes with bidirectional promoters share.

It has long been understood that selection for somatic alterations plays a critical role in carcinogenesis, but the complex landscape of mutations across cancers makes it difficult to understand the underlying process and why some mutations and not others get selected, or even which mutations may play a more important role in disease progression. In this work, we take another step forward in understanding this complex process, demonstrating how multi-layered constraints might affect selection, as tumor cells must still remain viable. Furthermore, we provide evidence that bidirectional promoters are an important genomic architecture that is protected from somatic alteration in addition to the germ line, as has been noted previously. Finally, our results suggest that when somatic alterations do occur in bidirectional promoters, particular notice should be paid, and the functional consequences of both genes in the pair should be considered.

## 4. Materials and Methods

### 4.1. Promoter Identification and Definitions

We defined a region as a bidirectional promoter if it fell between the TSSs of genes on opposite strands that are within 1000 bp of each other and extended this region to include 200 bp downstream of each TSS. We restricted our definition to exclude promoters with overlapping genes. Bidirectional promoters were then identified by querying the annotables package for R, which includes annotations for the GRCh37 version of the human genome obtained through Ensembl Biomart [26]. We then divided these promoters into two groups: bidirectional promoters between two coding genes (C/C bidirectional promoters) and bidirectional promoters between one coding and one non-coding gene (C/N bidirectional promoters). We did not use promoters between two noncoding genes. Single gene promoters were defined as the regions that are not bidirectional promoters, within 439 bp upstream and 200 bp downstream of a TSS, in order to make their mean width equal to that of the bidirectional promoters and avoid biasing the analysis by distance of alteration from promoter. Using the above definitions for promoters, 725 C/C bidirectional promoters, 135 C/N bidirectional promoters, and 17,639 single gene promoters were identified. For some analyses, we restricted the regions to those intersecting CpG islands. In such cases, this left 657 C/C bidirectional promoters, 97 C/N bidirectional promoters, and 5003 single gene promoters.

### 4.2. DNA Methylation Data

DNA methylation profiles were created by The Cancer Genome Atlas (TCGA) [27] using Illumina’s Infinium HumanMethylation450 BeadChip platform. Data for fourteen cancer types were obtained from the National Cancer Institute’s Genomic Data Commons Data Portal Legacy Archive [28] (Table 1). Data were functionally normalized [29] using the RnBeads package for R [30]. We used every TCGA dataset for which there were 10 or more matched tumor and normal samples. Differential methylation analysis was conducted using RnBeads and scored using the combined rank of differential methylation, as recommended by the authors. The combined rank is assigned as the maximum rank for differential methylation based on one of three methods: absolute difference in mean methylation level (by β-value [31]), absolute value of the log ratio of mean methylation level (by β-value), or *p*-value for differential methylation based on a linear model of the M-values (which have a distribution more amenable to linear models [31]) for the CpGs in tumor or tumor adjacent normal tissue. Overall differences in the combined rank of differential methylation between CpGs occurring in single gene and either C/C or C/N bidirectional promoters were then tested using a two-sided Wilcoxon rank sum test.

### 4.3. Simple Somatic Mutation Data

Simple somatic mutation (SSM) data were obtained through the International Cancer Genome Consortium’s Data Portal [32]. We downloaded all datasets containing SSMs found through whole genome sequencing. We examined differences in the odds of simple somatic mutations between single gene and bidirectional promoters using the count of SSMs in each promoter type. Each SSMs was counted only once, even if it spanned more than one base. Differences were tested using a logistic regression model of the log odds of SSM given promoter type.

### 4.4. Somatic Copy Number Alteration Data

Somatic copy number alteration data were downloaded through the Genomic Data Commons (GDC) Data Portal [28]. These data are processed through the GDC’s genomic harmonization pipelines that ensure all datasets are processed using the same workflows and are aligned to the GRCh38 Human reference genome. However, given that the rest of our analysis is based on the GRCh37 reference genome, we lifted over all copy number alteration coordinates to GRCh37 using the rtracklayer package for R [33]. We modelled the odds of promoters intersecting regions of copy number alteration for each promoter type using a logistic mixed effects model with a random intercept for each sample id. A segment was defined as having copy number gain if the segment mean was ≥5 and a copy number loss if it was ≤−75, where the segment mean is given as the log_2_ (n/2) and n is the mean copy number for a segment.

## Figures and Tables

**Table 1:** Methylation and copy number alteration datasets used in this work.

Cancer	Methylation Matched Tumor and Normal Samples	Promoter Probes	Copy Number Samples
Bladder Urothelial Carcinoma	21	37,532	412
Breast Invasive Carcinoma	96	37,124	1094
Colorectal Adenocarcinoma	37	37,088	614
Esophogeal Carcinoma	16	37,324	184
Head & Neck Squamous Cell Carcinoma	50	37,303	517
Kidney Renal Clear Cell Carcinoma	160	37,325	530
Kidney Papillary Carcinoma	45	37,227	290
Hepatocellular Carcinoma	49	36,845	375
Lung Adenocarcinoma	32	37,082	518
Lung Small Cell Carcinoma	42	37,581	503
Pancreatic Adenocarcinoma	10	37,365	184
Prostate Adenocarcinoma	50	37,416	497
Thyroid Carcinoma	56	37,779	505
Uterine Corpus Endometrial Carcinoma	46	37,296	540

**Table 2:** Simple somatic mutation datasets used in this work.

Cancer	ICGC Project Code	Samples	Countries
Chronic Lymphocytic Leukemia (ES)	CLLE-ES	201	Spain
Ductal Breast Carcinoma (EU/UK)	BRCA-EU	560	European Union, United Kingdom
Esophageal Adenocarcinoma (UK)	ESAD-UK	203	United Kingdom
Ewing Sarcoma (FR)	BOCA-FR	98	France
Malignant Lymphoma (DE)	MALY-DE	100	Germany
Ovarian Serous Cystadenocarcinoma (AU)	OV-AU	93	Australia
Pancreatic Adenocarcinoma (AU)	PACA-AU	252	Australia
Pancreatic Adenocarcinoma (CA)	PACA-CA	259	Canada
Pediatric Brain Cancer (DE)	PBCA-DE	380	Germany
Prostate Adenocarcinoma (CA)	PRAD-CA	124	Canada
Prostate Adenocarcinoma (UK)	PRAD-UK	108	United Kingdom
Renal Cell Carcinoma (EU/FR)	RECA-EU	95	European Union, France

**Table 3:** Enrichment of bidirectional vs. single gene promoters for DNAse hypersensitive sites.

Tissue Type	Promoter Type	Odds Ratio	95% CI	*p*-Value
Breast	C/C Bidirectional	23.73	[18.72, 30.43]	<2.20 × 10^−16^
	C/N Bidirectional	6.21	[4.27, 9.15]	<2.20 × 10^−16^
Pancreas	C/C Bidirectional	28.36	[21.53, 38.02]	<2.20 × 10^−16^
	C/N Bidirectional	6.53	[4.41, 9.86]	<2.20 × 10^−16^
Placenta	C/C Bidirectional	23.05	[18.30, 29.30]	<2.20 × 10^−16^
	C/N Bidirectional	7.02	[4.80, 10.41]	<2.20 × 10^−16^
Ovary	C/C Bidirectional	33.60	[23.38, 43.66]	<2.20 × 10^−16^
	C/N Bidirectional	10.42	[6.60, 17.14]	<2.20 × 10^−16^

**Table 4:** Enrichment of genes with C/C bidirectional promoters for gene ontology biological process terms.

Pathway	GO ID	Total	Observed	Expected	Ratio	adjP
DNA metabolic process	GO:0006259	899	138	63.98	2.16	0.00 × 10^0^
RNA processing	GO:0006396	851	130	60.56	2.15	0.00 × 10^0^
DNA repair	GO:0006281	472	87	33.59	2.59	3.11 × 10^−13^
chromosome organization	GO:0051276	562	95	39.99	2.38	2.33 × 10^−12^
ncRNA metabolic process	GO:0034660	535	91	38.07	2.39	5.41 × 10^−12^
ncRNA processing	GO:0034470	379	68	26.97	2.52	1.29 × 10^−9^
cellular response to DNA damage stimulus	GO:0006974	731	104	52.02	2	5.60 × 10^−9^
organelle fission	GO:0048285	578	84	41.13	2.04	2.10 × 10^−7^
mitochondrion organization	GO:0007005	599	86	42.63	2.02	2.10 × 10^−7^
cell cycle	GO:0007049	1591	178	113.22	1.57	2.10 × 10^−7^
double-strand break repair	GO:0006302	181	39	12.88	3.03	2.39 × 10^−7^
nuclear division	GO:0000280	537	79	38.21	2.07	2.87 × 10^−7^
cell cycle process	GO:0022402	1217	143	86.61	1.65	4.92 × 10^−7^
DNA recombination	GO:0006310	244	46	17.36	2.65	5.42 × 10^−7^
telomere maintenance	GO:0000723	119	29	8.47	3.42	1.69 × 10^−6^
telomere organization	GO:0032200	122	29	8.68	3.34	2.94 × 10^−6^
DNA conformation change	GO:0071103	235	43	16.72	2.57	4.03 × 10^−6^
nucleic acid phosphodiester bond hydrolysis	GO:0090305	264	46	18.79	2.45	5.68 × 10^−6^
DNA biosynthetic process	GO:0071897	187	36	13.31	2.71	1.53 × 10^−5^
rRNA metabolic process	GO:0016072	250	43	17.79	2.42	2.25 × 10^−5^
ribonucleoprotein complex biogenesis	GO:0022613	420	60	29.89	2.01	6.35 × 10^−5^
mitotic cell cycle process	GO:1903047	842	100	59.92	1.67	7.95 × 10^−5^
ribosome biogenesis	GO:0042254	302	47	21.49	2.19	1.07 × 10^−4^
mRNA processing	GO:0006397	442	61	31.45	1.94	1.49 × 10^−4^
rRNA processing	GO:0006364	243	40	17.29	2.31	1.72 × 10^−4^
mitotic cell cycle	GO:0000278	926	106	65.9	1.61	1.76 × 10^−4^
mitotic nuclear division	GO:0007067	411	57	29.25	1.95	2.70 × 10^−4^
mRNA metabolic process	GO:0016071	631	78	44.9	1.74	3.11× 10^−4^
chromatin organization	GO:0006325	676	82	48.11	1.7	3.41 × 10^−4^
tRNA processing	GO:0008033	115	24	8.18	2.93	4.10 × 10^−4^
DNA synthesis involved in DNA repair	GO:0000731	71	18	5.05	3.56	4.31 × 10^−4^
mitochondrial translation	GO:0032543	117	24	8.33	2.88	5.28 × 10^−4^
DNA-templated transcription, termination	GO:0006353	94	21	6.69	3.14	5.28 × 10^−4^
chromosome segregation	GO:0007059	305	45	21.7	2.07	5.64 × 10^−4^
cellular macromolecular complex assembly	GO:0034622	876	99	62.34	1.59	5.64 × 10^−4^
protein folding	GO:0006457	204	34	14.52	2.34	6.34 × 10^−4^
regulation of chromosome organization	GO:0033044	128	25	9.11	2.74	7.25 × 10^−4^
regulation of organelle organization	GO:0033043	963	106	68.53	1.55	7.58 × 10^−4^
DNA packaging	GO:0006323	155	28	11.03	2.54	9.00 × 10^−4^
mitochondrial translational elongation	GO:0070125	83	19	5.91	3.22	9.01 × 10^−4^
